# Human T cell leukemia virus type 1 (HTLV-1) and oncogene or oncomiR addiction?

**DOI:** 10.18632/oncotarget.179

**Published:** 2010-10-01

**Authors:** Kuan-Teh Jeang

**Affiliations:** ^1^ Laboratory of Molecular Microbiology, National Institute of Allergy and Infectious Diseases, the National Institutes of Health, Bethesda, Maryland, USA 20892

**Keywords:** HTLV, ATL, MicroRNAs, Leukemia, oncogene

## Abstract

The mechanism of HTLV-1 transformation of cells to Adult T cell leukemia (ATL) remains not fully understood. Currently, the viral Tax oncoprotein is known to be required to initiate transformation. Emerging evidence suggests that Tax is not needed to maintain the transformed ATL phenotype. Recent studies have shown that HTLV-1 transformed cells show deregulated expression of cellular microRNAs (miRNAs). Here we discuss the possibility that early ATL cells are Tax-oncogeneaddicted while late ATL cells are oncogenic microRNA (oncomiR) – addicted. The potential utility of interrupting oncomiR addiction as a cancer treatment is broached.

HTLV-1 was the first human retrovirus to be isolated. It was identified in 1980 by Robert Gallo and co-workers [1]; that initial finding was followed closely by important contributions from Japanese virologists [2]. HTLV-1 is causative of Adult T cell leukemia [3,4], a treatment refractory T cell cancer found endemically in Japan [5] and elsewhere [6]. Studies on this virus over the past three decades have provided insight into oncogene- and oncogenic microRNA- (oncomiR) addiction in leukemic transformation.

HTLV-1 encodes a viral Tax oncoprotein [7-9] whose expression confers prosurvival and proproliferative properties to infected cells. Extant findings have shown that Tax is sufficient to transform human T cells [10,11]. Hence, the expression of Tax-alone in transgenic mice was found to be fully proficient for *in vivo* tumorigenesis [12-14]. Indeed, current data are consistent with the notion that Tax expression in infected humans greatly accelerates the *in vivo* cycling of T cells [15]. Intriguingly, when ATL patients are followed over time, a puzzling finding reveals that Tax expression *in vivo* is absent from approximately 60% of late leukemias [16]. Thus, unlike other virus-induced human malignancies such as the cervical cancers caused by human papilloma virus (HPV), in which the expression of the viral E6 and E7 oncoproteins are required for tumor maintenance [17], late ATL cells are apparently not addicted to the Tax oncoprotein. Why might ATL cells extinguish Tax expression? A possible reason is because this viral protein represents the major target for cytotoxic T-lymphocytes (CTL) in infected patients [18,19]. Accordingly, the loss of Tax expression *in vivo* would facilitate the escape of virus-infected cells from CTL surveillance; and this seemingly would benefit disease progression.

A currently accepted model for ATL genesis by HTLV-1 is that the viral Tax oncogene is used for the initiation, but not the maintenance, of leukemogenesis (Figure 1). In this regard, the HTLV-1 – ATL transformation mechanism appears not to subscribe to the oncogene addiction model of carcinogenesis [20]. What might then be some of the factor(s) needed for ATL cells to maintain their leukemic phenotype in the absence of Tax? One possible explanation rests with the observation that all ATL cells exhibit virus-mediated attenuation of the cell's spindle assembly checkpoint [21] and are thus highly aneuploid [9]. Potentially, this selected presentation of aneuploid chromosomes could be sufficient per se for maintaining the transformed ATL phenotype [22]. A second possibility is that transformed ATL cells have acquired altered expression of cellular microRNAs that are capable, in a Tax-independent fashion, of maintaining oncogenesis (e.g. oncomiRs [23] [24]).

**Figure 1:**
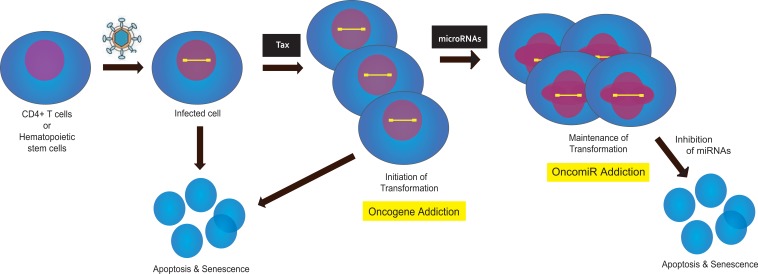
Potential stages of oncogene-addiction and oncomiR-addiction in HTLV-1 transformation of ATL leukemic T cells Virus-infected cells either initiate transformation after Tax expression or enter apoptosis/senescence. At this stage the cells could be regarded as Tax-oncogene-addicted. Subsequently, the expression of Tax in ATL cells is extinguished, and maintenance of the transformed phenotype in the cells is postulated to emerge from altered miRNA expression (oncomiR-addiction). Inhibition of the activity of oncomiRs can send such cells in tissue culture into apoptosis/senescence. (The figure is modified from Jeang, KT, *JFMA*, 2010, in press).

Altered miRNA expression has indeed been linked to carcinogenesis. Early on, it was found that the loss of miR-15a and miR-16-1 correlated with B-cell chronic lymphocytic leukemia [25]. Later, miRNA signatures for various cancers were described and linked to oncogenic transformation and found to be diagnostic of tumor types [23,26]. The deregulated expression of miRNAs in HTLV-1 transformed cells has also been reported in three independent publications [27-29]. In parsing the specific miRNA changes published in the three HTLV-1 studies, there appears to be very little over lap amongst most of the miRNA moieties [30]. Nonetheless, there was an intriguing consensus amongst the three findings. For example, in the study by Yeung *et al*., the authors reported that the tumor suppressor protein TP53INP1 in HTLV-1- infected/transformed cells was targeted for repression by the upregulated expression of miR-93 and miR-130b [27]. By comparison, in the subsequent study by Pichler *et al*., TP53INP1 was also reported to be targeted in HTLV-1 infected/transformed cells, but by the upregulated expression of miR-21, -24, -146a, and -155 [28]. Remarkably, separate from the in vitro HTLV-1 infected/transformed cells, Bellon et al. and Yeung et al. further investigated *in vivo* ATL leukemic cells from patients; and both noted upregulated miR-155 expression [27,29] which would be consistent with a silencing of TP53INP1 by miR-155 [31]. Thus, collectively, the three studies agree and converge on TP53INP1 as one of the important miRNA-regulated targets in ATL transformation by HTLV-1.

Based on the above data, one biological scenario is that late ATL cells may indeed be oncomiR-addicted while early ATL cells are Tax-oncogene-addicted (Figure 1). Recently, Watashi et al. have provided additional evidence that NIH 3T3 mouse cells can be transformed by singular over expression of either miR-93 or miR-130b [32]. They discovered two small molecule compounds that can be used to reduce the over expression of miR-93 or miR-130b, and they showed that the treatment of miR-93- or miR-130b transformed NIH 3T3 cells using such compounds reversed tumorigenesis [32]. These results support the interpretation that in certain settings oncomiR-addicted tumors exist, and that this addiction could represent a potential treatment target for such cancers.

One might reason that a logical extension is to treat cancers by reducing oncomiR expression as well as targeting oncogene expression. Reality may be more complicated than this simple logic. Some studies have shown that a generalized down regulation of miRNAs is frequently seen in human cancers [26,33]. While it is not fully understood how general miRNA down regulations could propitiate carcinogenesis, such observations do raise caution that small molecule inhibitors of oncomiR activity needs to be utilized judiciously and monitored carefully to ensure that they ameliorate rather than exacerbate cancers. Further investigations are needed to conclusively verify oncomiR inhibition as an important treatment option in cancers.
